# A successful in vitro fertilization outcome in a hermaphrodite male

**DOI:** 10.1002/ijgo.16039

**Published:** 2024-11-18

**Authors:** Shima Elbakhit M. E. Albasha, Sami Saadi Al‐Said, Haitham Tharwat M. H. El Bardisi

**Affiliations:** ^1^ Department of Obstetrics and Gynecology Hamad Medical Corporation Doha Qatar; ^2^ Department of Urology Hamad Medical Corporation Doha Qatar

**Keywords:** bilateral gonadopexy, in vitro fertilization, infertility, male hermaphrodism, orchidopexy, ovotesticular syndrome

## Abstract

A case of male hermaphroditism who underwent surgery for the removal of female adnexa and bilateral gonadopexy at 2 years of age is presented.

## 1 CASE REPORT

Ovotesticular syndrome or hermaphroditism, an intersex condition in which one or both gonads are ovotestis, could be true or pseudo‐hermaphroditism.^1^ True hermaphroditism is reported to be 1/100000 with external genitalia varying per gender. Pseudo‐hermaphroditism has gonads of one sex and genital ducts or external genitalia of the other and is more frequent.^2^ In male hermaphroditism, there may be the need for assisted conception. A rare case of pregnancy in a male hermaphrodite is presented.

The patient provided consent for publication, and ethical approval was obtained from the Medical Research Center Team with approval number MRC‐04‐24‐254. This was a case of a 28‐year‐old with hermaphroditism, a person who subsequently grew and socialized as a male, with a history of laparoscopic exploration laparotomy, and excision of female adnexa and bilateral gonadopexy at 2 years of age. He had no other medical history of note. His wife was 25 years of age, with irregular menstrual cycles. Had no medical or surgical history.

In 2021, the couple visited the In Vitro Fertilization Clinic at Hamad Medical Corporation due to primary infertility after 6 years of marriage. The weight of the female was 52 kg and her body mass index was 20 (calculated as weight in kilograms divided by the square of height in meters). The weight of the male was 90 kg, and he had a normal phallus and no gynecomastia. At 2 years of age, he underwent bilateral orchidopexy for bilateral undescended testis and excision of female adnexa and Müllerian duct remnants.

On physical examination, only the left testis was felt. Semen analysis showed hypospermia; the semen volume was <0.5 mL and was alkaline, azoospermic, and with normal viscosity and liquefication. On ultrasound, the right testis was not visualized in the scrotal sac. An oval‐shaped solid structure, which was atophic testis, was observed in the right scrotal sac with an approximate measure of 10 × 7 cm. The measurement of the left testis was 4.6 × 3.5 × 2 cm and approximately 21 cm^3^. Normal vascularity and echotexture were observed on the color Doppler study. The left epididymis was also of normal size, texture, and vascularity. An epididymal head cyst was noted and measured at 12 mm.

## 2 ADDITIONAL DETAILS

The husband underwent testicular sperm aspiration (TESA) in 2021; three aspirations were done and sent for intracytoplasmic sperm injection (ICSI) and freezing. The in vitro fertilization (IVF) cycle was initiated on 10 October 2021, using a long protocol. Stimulation was started using Gonal F 112.5 for a duration of 7 days, triggered with a human chorionic gonadotropin (hCG) injection 10 000. Transvaginal ultrasound was done on January 31, 2022 and the post‐embryo transfer was done on January 3, 2022. Stimulation was stopped on October 31, 2021. No Gonal F was given on this day. Gonal F (11.25 IU) was given only for 7 days. More than 10 follicles were developed. The right ovary follicle sizes were 22, 20, 18, 14, 14, 15, and 14 mm and the left ovary follicle sizes were 20, 19, 17, 16, 16, and 16 mm. A total of 21 eggs were collected; 15 were mature and 9 were fertilized. On day 5, six frozen embryos, and on day 6 three frozen embryos were developed. All embryos were frozen due to ovarian hyperstimulation syndrome and no pre‐implantation genetic testing was done. For endometrial preparation, hormonal replacement therapy (HRT) was given with oral estradiol tablet for 8 days and progesterone gel 8% + dydrogesterone 10 mg two times per day.

In January 2022, one frozen embryo was transferred, after 2 weeks (January 17, 2022) beta‐hCG was checked which showed a positive result of 1316 mIU/mL. The ultrasound showed a single viable pregnancy and the patient was referred to an antenatal care clinic. The pregnancy was uneventful. On September 22, 2022, vacuum delivery was performed, and a healthy male was delivered weighing 3094 g, with a gestational age of 40 weeks +1 day.

The patient underwent frozen embryo transfer on February 5, 2024, with two embryos transferred. The patient was pregnancy‐positive with 13 weeks gestation and 2 days.

We present here a case of hermaphroditism with a successful pregnancy via IVF and delivery, and to the best of our knowledge, this is the first report of a successful pregnancy through IVF for a case of male hermaphroditism in Qatar. Since 1978, the year when the first successful IVF pregnancy was reported,^3^ IVF techniques have vastly improved to give hope to many who are suffering from infertility.^4^ The first case of true hermaphroditism was reported in 1955 by Swyer in a case report of two women with a 46 XX karyotype.^5^ Decades later, the pathogenesis and the etiology remain unclear.

Hermaphroditism can be caused by the division of one ovum, fertilization of each haploid ovum, and fusion of the two zygotes. An ovum can also be fertilized by two sperm and thereafter trisomic rescue in one or more daughter cells. If one male zygote and one female zygote fuse, two ova fertilized by two sperm cells will occasionally fuse to form a tetragametic chimera. A mutation in the SRY gene has been linked to it.^2^ Although rare, more than 200 cases have been reported in the literature. XX is by far the most common karyotype; most patients with this form are SRY‐negative, but despite this, they can develop testicular and ovarian tissue.^6^ For such patients, the female sex of rearing appears to be the most appropriate, and it is critical to try to preserve the ovarian portion of the ovotestis in these cases, allowing the patient to have normal puberty and fertility.^7^

In a review by Krob et al., 97% of 283 hermaphrodites studied showed a 46 XX karyotype.^6^ The karyotypes of these cases are 46 XX, or 46 XY, or a mosaic 46 XX/46 XY. Narita et al. reported a case of a 25‐year‐old hermaphrodite who, after surgery, later married and delivered a normal male via cesarean section. Zayed et al. reported a case of a 41‐year‐old male hermaphrodite who was treated for seminoma and who later fathered a healthy child via IVF‐ICSI. Sugawara et al. reported on a 46‐year‐old hermaphrodite with a 46 XX/46 XY karyotype who later fathered a healthy male using IVF. A successful second delivery was even reported in 2012. In summary, to the best of our knowledge, this is the first report of a successful pregnancy through IVF for a case of male hermaphroditism in Qatar.

After the reported case, at the time of manuscript submission, the patient had embarked on her second pregnancy.

## AUTHOR CONTRIBUTIONS

Shima Elbakhit M. E. Albasha, Sami Saadi Al‐Said, and Haitham Tharwat M. H. El Bardisi conceived and designed the study. Shima Elbakhit M. E. Albasha and Haitham Tharwat M. H. El Bardisi wrote and critically edited the paper. All authors approved the final version of the paper.

## FUNDING INFORMATION

This study was not funded by anyone or any organization.

## CONFLICT OF INTEREST STATEMENT

The authors have no conflicts of interest.

## Data Availability

Data sharing is not applicable to this article as no new data were created or analyzed in this study.
